# Supplementary material for: Lotka-Volterra pairwise modeling fails to capture diverse pairwise microbial interactions
Source: eLife. 2017 Mar 28;6:e25051. doi: 10.7554/eLife.25051 (PMC5469619; doi:10.7554/eLife.25051)
Supplement: Figure 3—source data 2. — DOI: http://dx.doi.org/10.7554/eLife.25051.009 [file elife-25051-fig3-data2.docx]

%% A: case II: a steady-state exists, R_S_(0)=0.8, S_10_=1e3

r0 = [0.12; 0.1]; % population reproduction rates, per hour

at = 0.05; % avg. consumption values (fmole per cell); alpha_ij: population i, resource j

bt = 0.1; % avg. production rates (fmole per cell per hour); beta_ij: population i, resource j

K1 = 1e5; % K_C1S2, Michaelis-Menten coefficient for influence, fmole/ml

K2 = 1e5; % K_S2C1, Michaelis-Menten coefficient for consumption, fmole/ml

S10=1e5 % initial number of S1 cell

S20=0.8e5 % initial number of S2 cell

%% B: case II: a steady-state exists, R_S_(0)=4, S_10_=1e5

r0 = [0.12; 0.1]; % population reproduction rates, per hour

at = 0.05; % avg. consumption values (fmole per cell); alpha_ij: population i, resource j

bt = 0.1; % avg. production rates (fmole per cell per hour); beta_ij: population i, resource j

K1 = 1e5; % K_C1S2, Michaelis-Menten coefficient for influence, fmole/ml

K2 = 1e5; % K_S2C1, Michaelis-Menten coefficient for consumption, fmole/ml

S10=1e5 % initial number of S1 cell

S20=4e5 % initial number of S2 cell

%% C: case II: a steady-state exists, R_S_(0)=0.2, S_10_=1e5

r0 = [0.12; 0.1]; % population reproduction rates, per hour

at = 0.05; % avg. consumption values (fmole per cell); alpha_ij: population i, resource j

bt = 0.1; % avg. production rates (fmole per cell per hour); beta_ij: population i, resource j

K1 = 1e5; % K_C1S2, Michaelis-Menten coefficient for influence, fmole/ml

K2 = 1e5; % K_S2C1, Michaelis-Menten coefficient for consumption, fmole/ml

S10=1e5 % initial number of S1 cell

S20=0.2e5 % initial number of S2 cell

%% D: case II: a steady-state exists, R_S_(0)=0.8, S_10_=1e3

r0 = [0.12; 0.1]; % population reproduction rates, per hour

at = 0.05; % avg. consumption values (fmole per cell); alpha_ij: population i, resource j

bt = 0.1; % avg. production rates (fmole per cell per hour); beta_ij: population i, resource j

K1 = 1e5; % K_C1S2, Michaelis-Menten coefficient for influence, fmole/ml

K2 = 1e5; % K_S2C1, Michaelis-Menten coefficient for consumption, fmole/ml

S10=1e3 % initial number of S1 cell

S20=0.8e3 % initial number of S2 cell

%% E: case III, no steady-state exists, R_S_(0)=1, S_10_=5e4

r0 = [0.1; 0.09]; % population reproduction rates, per hour

at = 0.05; % avg. consumption values (fmole per cell); alpha_ij: population i, resource j

bt = 0.1; % avg. production rates (fmole per cell per hour); beta_ij: population i, resource j

K1 = 1e5; % K_C1S2, Michaelis-Menten coefficient for influence, fmole/ml

K2 = 1e5; % K_S2C1, Michaelis-Menten coefficient for consumption, fmole/ml

S10=5e4 % initial number of S1 cell

S20=5e4 % initial number of S2 cell

%% F: case III, no steady-state exists, R_S_(0)=10, S_10_=1e4

r0 = [0.1; 0.09]; % population reproduction rates, per hour

at = 0.05; % avg. consumption values (fmole per cell); alpha_ij: population i, resource j

bt = 0.1; % avg. production rates (fmole per cell per hour); beta_ij: population i, resource j

K1 = 1e5; % K_C1S2, Michaelis-Menten coefficient for influence, fmole/ml

K2 = 1e5; % K_S2C1, Michaelis-Menten coefficient for consumption, fmole/ml

S10=1e4 % initial number of S1 cell

S20=1e5 % initial number of S2 cell

%% G: case III, no steady-state exists, R_S_(0)=0.25, S_10_=8e4

r0 = [0.1; 0.09]; % population reproduction rates, per hour

at = 0.05; % avg. consumption values (fmole per cell); alpha_ij: population i, resource j

bt = 0.1; % avg. production rates (fmole per cell per hour); beta_ij: population i, resource j

K1 = 1e5; % K_C1S2, Michaelis-Menten coefficient for influence, fmole/ml

K2 = 1e5; % K_S2C1, Michaelis-Menten coefficient for consumption, fmole/ml

S10=8e4 % initial number of S1 cell

S20=2e4 % initial number of S2 cell

%% H: case III, no steady-state exists, R_S_(0)=10, S_10_=1e2

r0 = [0.1; 0.09]; % population reproduction rates, per hour

at = 0.05; % avg. consumption values (fmole per cell); alpha_ij: population i, resource j

bt = 0.1; % avg. production rates (fmole per cell per hour); beta_ij: population i, resource j

K1 = 1e5; % K_C1S2, Michaelis-Menten coefficient for influence, fmole/ml

K2 = 1e5; % K_S2C1, Michaelis-Menten coefficient for consumption, fmole/ml

S10=1e2 % initial number of S1 cell

S20=1e3 % initial number of S2 cell
