# Supplementary material for: Lotka-Volterra pairwise modeling fails to capture diverse pairwise microbial interactions
Source: eLife. 2017 Mar 28;6:e25051. doi: 10.7554/eLife.25051 (PMC5469619; doi:10.7554/eLife.25051)
Supplement: Figure 3—source data 3. — DOI: http://dx.doi.org/10.7554/eLife.25051.010 [file elife-25051-fig3-data3.docx]

**Fig.3-FS3(A)**

r0 = [0.12; 0.1]; % population reproduction rates of S_1_ and S_2_, per hour

K2 = 1e5; % K_S2C1, Michaelis-Menten coefficient for consumption, fmole/ml

K1 = 2e5; % K_C1S2, Michaelis-Menten coefficient for consumption, fmole/ml

alpha = 0.05; % avg. consumption values (fmole per cell);

beta = 0.1; % avg. production rates (fmole per cell per hour);

rint = [0; 0.05]; % Nc*Nm matrix of interaction coefficients

**Fig.3-FS3(B)**

r0 = [0.12; 0.1]; % population reproduction rates of S_1_ and S_2_, per hour

K2 = 2e5; % K_S2C1, Michaelis-Menten coefficient for consumption, fmole/ml

K1 = 1e5; % K_C1S2, Michaelis-Menten coefficient for influence, fmole/ml

alpha = 0.05; % avg. consumption values (fmole per cell);

beta = 0.1; % avg. production rates (fmole per cell per hour);

rint = [0; 0.05]; % Nc*Nm matrix of interaction coefficients

**Fig.3-FS3(C)**

r0 = [0.08; 0.1]; % population reproduction rates of S_1_ and S_2_, per hour

K2 = 1e5; % K_S2C1, Michaelis-Menten coefficient for consumption, fmole/ml

K1 = 2e5; % K_C1S2, Michaelis-Menten coefficient for influence, fmole/ml

alpha = 0.05; % avg. consumption values (fmole per cell);

beta = 0.1; % avg. production rates (fmole per cell per hour);

rint = [0; 0.05]; % Nc*Nm matrix of interaction coefficients

**Fig.3-FS3(D)**

r0 = [0.07; 0.1]; % population reproduction rates of S_1_ and S_2_, per hour

K2 = 1e6; % K_S2C1, Michaelis-Menten coefficient for consumption, fmole/ml

K1 = 1e5; % K_C1S2, Michaelis-Menten coefficient for influence, fmole/ml

alpha = 0.05; % avg. consumption values (fmole per cell);

beta = 0.1; % avg. production rates (fmole per cell per hour);

rint = [0; 0.05]; % Nc*Nm matrix of interaction coefficients
