# Supplementary material for: Lotka-Volterra pairwise modeling fails to capture diverse pairwise microbial interactions
Source: eLife. 2017 Mar 28;6:e25051. doi: 10.7554/eLife.25051 (PMC5469619; doi:10.7554/eLife.25051)
Supplement: Figure 5—source data 2. — DOI: http://dx.doi.org/10.7554/eLife.25051.022 [file elife-25051-fig5-data2.docx]

%% Fig 5-FS1B

% window 1

r0 = [0.13; 0.12]; % population reproduction rates, per hour

CSD = 1e2; % total initial cells

K = 1e5; % Michaelis-Menten coefficient, fmole/ml

ExtTh = 0.1; % population extinction threshold

DilTh = 1e5; % coculture dilution threshold

tau0 = 0;

tauf = 250; % in hours

dtau = 0.01; % in hours, cell growth update and uptake timescale

at = 0.1; % avg. consumption values (fmole per cell); alpha_ij: population i, resource j

bt = 0.1; % avg. production rates (fmole per cell per hour); beta_ij: population i, resource j

rint = [0 0; 0.04 -0.05]; % Nc*Nm matrix of interaction coefficients

[Nc Nm] = size(rint);

KMM = K*[1 0.01; 1 1]; % Nm*Nc, Michaelis-Menten coefficients for consumption and influence, fmole/ml

rp0 = 1/Nc*ones(1,Nc);

% receiving matrix, Nm x Nc

R = [0 1; 0 1];

% producing matrix, Nm x Nc

P = [1 0; 1 0];

% interaction matrix

alpha = at*[0 1; 0 1]; % consumption rates

beta = bt*[1 0; 1 0]; % mediator release rates

A = (R.*alpha)';

B = (P.*beta)';

% window 2

r0 = [0.13; 0.12]; % population reproduction rates, per hour

CSD = 1e6; % total initial cells

K = 1e5; % Michaelis-Menten coefficient, fmole/ml

ExtTh = 0.1; % population extinction threshold

DilTh = 1e9; % coculture dilution threshold

tau0 = 0;

tauf = 250; % in hours

dtau = 0.01; % in hours, cell growth update and uptake timescale

at = 0.1; % avg. consumption values (fmole per cell); alpha_ij: population i, resource j

bt = 0.1; % avg. production rates (fmole per cell per hour); beta_ij: population i, resource j

rint = [0 0; 0.04 -0.05]; % Nc*Nm matrix of interaction coefficients

[Nc Nm] = size(rint);

KMM = K*[1 0.01; 1 1]; % Nm*Nc, Michaelis-Menten coefficients for consumption and influence, fmole/ml

rp0 = 1/Nc*ones(1,Nc);

% receiving matrix, Nm x Nc

R = [0 1; 0 1];

% producing matrix, Nm x Nc

P = [1 0; 1 0];

% interaction matrix

alpha = at*[0 1; 0 1]; % consumption rates

beta = bt*[1 0; 1 0]; % mediator release rates

A = (R.*alpha)';

B = (P.*beta)';

%% Fig 5-FS1C; other parameters similar to Fig 5-FS1B

% Comparable potency

rint = [0 0; 0.04 -0.05]; % Nc*Nm matrix of interaction coefficients

KMM = K*[1 0.09; 1 0.12]; % Nm*Nc, Michaelis-Menten coefficients for consumption and influence, fmole/ml

%% Fig 5-FS1D; other parameters similar to Fig 5-FS1B

% One mediator dominant

rint = [0 0; 0.002 -0.05]; % Nc*Nm matrix of interaction coefficients

KMM = K*[1 0.01; 1 1]; % Nm*Nc, Michaelis-Menten coefficients for consumption and influence, fmole/ml
