# Supplementary material for: Lotka-Volterra pairwise modeling fails to capture diverse pairwise microbial interactions
Source: eLife. 2017 Mar 28;6:e25051. doi: 10.7554/eLife.25051 (PMC5469619; doi:10.7554/eLife.25051)
Supplement: Figure 6—source data 1. — DOI: http://dx.doi.org/10.7554/eLife.25051.025 [file elife-25051-fig6-data1.docx]

**%% Part B – coexistence**

r0 = [-0.03; -0.05]; % population reproduction rates, per hour

CSD = 10; % total initial cells

K = 1e5; % Michaelis-Menten coefficient, fmole/ml

ExtTh = 1e-16;

DilTh = 1e16; % coculture dilution threshold

tau0 = 0;

tauf = 300; % in hours

dtau = 0.01; % in hours, cell growth update and uptake timescale

at = 1; % avg. consumption values (fmole per cell); alpha_ij: population i, resource j

bt = 1; % avg. production rates (fmole per cell per hour); beta_ij: population i, resource j

Nr = 1; % number of rounds of propagation

KC = K*[1.1 0;

1 1];

rc = [10; 13]; % Nc*1 matrix of resource-mediated fitness benefit

Nc = length(r0);

Nm = length(KC);

rp0 = 1/Nc*ones(1,Nc);

%% Connectivity, Nm*Nc

P = [1 1; 0 1]; % consumption

R = [0 0; 1 0]; % release

%% Rates, Nm*Nc

alpha = at*P; % consumption rates

beta = bt*[0 0; 1 0]; % mediator release rates

beta0 = bt*[100 0]; % resource supply rates

%% interaction matrix, Nc*Nm

A = (P.*alpha)'; % consumption matrix

B = (R.*beta)'; % release matrix

**%% Part C – exclusion**

r0 = [-0.03; -0.05]; % population reproduction rates, per hour

CSD = 10; % total initial cells

K = 1e5; % Michaelis-Menten coefficient, fmole/ml

ExtTh = 1e-16;

DilTh = 1e16; % coculture dilution threshold

tau0 = 0;

tauf = 300; % in hours

dtau = 0.01; % in hours, cell growth update and uptake timescale

at = 1; % avg. consumption values (fmole per cell); alpha_ij: population i, resource j

bt = 1; % avg. production rates (fmole per cell per hour); beta_ij: population i, resource j

Nr = 1; % number of rounds of propagation

KC = K*[1 0;

1 1]; % Nm*Nc, Kij values

rc = [12; 9]; % Nc*1 matrix of resource-mediated fitness benefit

Nc = length(r0);

Nm = length(KC);

rp0 = 1/Nc*ones(1,Nc);

%% Connectivity, Nm*Nc

P = [1 1; 0 1]; % consumption

R = [0 0; 1 0]; % release

%% Rates, Nm*Nc

alpha = at*P; % consumption rates

beta = bt*[0 0; 1 0]; % mediator release rates

beta0 = bt*[100 0]; % resource supply rates

%% interaction matrix, Nc*Nm

A = (P.*alpha)'; % consumption matrix

B = (R.*beta)'; % release matrix

**%% Part D – resurgence**

r0 = [-0.02; -0.01]; % population reproduction rates, per hour

CSD = 10; % total initial cells

K = 1e5; % Michaelis-Menten coefficient, fmole/ml

ExtTh = 1e-16;

DilTh = 1e16; % coculture dilution threshold

tau0 = 0;

tauf = 3000; % in hours

dtau = 0.01; % in hours, cell growth update and uptake timescale

at = 1; % avg. consumption values (fmole per cell); alpha_ij: population i, resource j

bt = 0.1; % avg. production rates (fmole per cell per hour); beta_ij: population i, resource j

Nr = 1; % number of rounds of propagation

KC = K*[1 0;

2 1]; % Nc*Nm

rc = [15; 25]; % Nc*1 matrix of resource-mediated fitness benefit

Nc = length(r0);

Nm = length(KC);

rp0 = 1/Nc*ones(1,Nc);

%% Connectivity, Nm*Nc

P = [1 1; 0 1]; % consumption

R = [0 0; 1 0]; % release

%% Rates, Nm*Nc

alpha = at*P; % consumption rates

beta = bt*[0 0; 10 0]; % mediator release rates

beta0 = bt*[10 0]; % resource supply rates

%% interaction matrix, Nc*Nm

A = (P.*alpha)'; % consumption matrix

B = (R.*beta)'; % release matrix
