# Supplementary material for: Lotka-Volterra pairwise modeling fails to capture diverse pairwise microbial interactions
Source: eLife. 2017 Mar 28;6:e25051. doi: 10.7554/eLife.25051 (PMC5469619; doi:10.7554/eLife.25051)
Supplement: Figure 7—source data 1. — DOI: http://dx.doi.org/10.7554/eLife.25051.027 [file elife-25051-fig7-data1.docx]

r0 = [0.14; 0.12; 0.11]; % population reproduction rates, per hour

CSD = 1e5; % total initial cells

K = 1e5; % Michaelis-Menten coefficient, fmole/ml

ExtTh = 0.01; % population extinction threshold

DilTh = 1e8; % coculture dilution threshold

tau0 = 0;

tauf = 250; % in hours

dtau = 0.01; % in hours, cell growth update and uptake timescale

at = 0; % avg. consumption values (fmole per cell); alpha_ij: population i, resource j

bt = 0.1; % avg. production rates (fmole per cell per hour); beta_ij: population i, resource j

Ng = 50;

Nr = round(Ng/(log(DilTh/CSD)/log(2)))+1; % number of rounds of propagation

rint = [0 0; 0.1 0; 0 0.07]; % Nc*Nm matrix of interaction coefficients

[Nc Nm] = size(rint);

KMM = K*[1 1 1; 1 1 1]; % Nm*Nc, Michaelis-Menten coefficients for consumption and influence, fmole/ml

rp0 = 1/3*[1 1 1]; %1/Nc*ones(1,Nc);

%% Parameters

% receiving matrix, Nm x Nc

R = [0 1 0; 0 0 1];

% producing matrix, Nm x Nc

P = [1 0 0; 0 1 0];

% interaction matrix

alpha = at*[0 1 0; 0 0 1]; % consumption rates

beta = bt*[1 0 0; 0 0.8 0]; % mediator release rates

A = (R.*alpha)';

B = (P.*beta)';
