# Supplementary material for: Lotka-Volterra pairwise modeling fails to capture diverse pairwise microbial interactions
Source: eLife. 2017 Mar 28;6:e25051. doi: 10.7554/eLife.25051 (PMC5469619; doi:10.7554/eLife.25051)
Supplement: Figure 7—source data 4. — DOI: http://dx.doi.org/10.7554/eLife.25051.030 [file elife-25051-fig7-data4.docx]

r0 = [0.135; 0.1; 0.11]; % population reproduction rates, per hour

CSD = 1e5; % total initial cells

K = 3e5; % Michaelis-Menten coefficient, fmole/ml

ExtTh = 0.1; % population extinction threshold

DilTh = 1e8; % coculture dilution threshold

tau0 = 0;

tauf = 250; % in hours

dtau = 0.01; % in hours, cell growth update and uptake timescale

at = 0; % avg. consumption values (fmole per cell); alpha_ij: population i, resource j

bt = 0.04; % avg. production rates (fmole per cell per hour); beta_ij: population i, resource j

Ng = 50; % number of generations for preconditioning (before fitting)

Nr = round(Ng/(log(DilTh/CSD)/log(2)))+1; % number of rounds of propagation

rint = [0; 0.11; 0.1]; % Nc*Nm matrix of interaction coefficients

[Nc Nm] = size(rint);

KMM = K*[1 0.5 1]; % Michaelis-Menten coefficients, fmole/ml

rp0 = 1/Nc*ones(1,Nc);

%% Parameters

R = [0 1 1];

P = [1 0 0];

% interaction matrix

alpha = at*[0 0 1]; % consumption rates

beta = bt*[1 0 0]; % mediator release rates

A = (R.*alpha)';

B = (P.*beta)';
