# Supplementary material for: Lotka-Volterra pairwise modeling fails to capture diverse pairwise microbial interactions
Source: eLife. 2017 Mar 28;6:e25051. doi: 10.7554/eLife.25051 (PMC5469619; doi:10.7554/eLife.25051)
Supplement: Figure 7—source data 5. — DOI: http://dx.doi.org/10.7554/eLife.25051.031 [file elife-25051-fig7-data5.docx]

r0 = [0.11; 0.08; 0.105]; % population reproduction rates, per hour

CSD = 1e5; % total initial cells

K = 1e9; % Michaelis-Menten coefficient, fmole/ml

ExtTh = 0.1; % population extinction threshold

DilTh = 1e8; % coculture dilution threshold

tau0 = 0;

tauf = 250; % in hours

dtau = 0.01; % in hours, cell growth update and uptake timescale

at = 0; % avg. consumption values (fmole per cell); alpha_ij: population i, resource j

bt = 0.04; % avg. production rates (fmole per cell per hour); beta_ij: population i, resource j

rint = [0; 7; 0]; % Nc*Nm matrix of interaction coefficients

KMM = K*[1 1 1]; % Michaelis-Menten coefficients, fmole/ml

%% Parameters

R = [0 1 0];

P = [1 0 1];

% interaction matrix

alpha = at*[0 0 0]; % consumption rates

beta = bt*[1 0 0.8]; % mediator release rates

A = (R.*alpha)';

B = (P.*beta)';
